# Current Progress of Mitochondrial Quality Control Pathways Underlying the Pathogenesis of Parkinson's Disease

**DOI:** 10.1155/2019/4578462

**Published:** 2019-08-14

**Authors:** Xue Jiang, Tao Jin, Haining Zhang, Jing Miao, Xiuzhen Zhao, Yana Su, Ying Zhang

**Affiliations:** Department of Neurology and Neuroscience Center, First Hospital of Jilin University, Xinmin Street No. 71, Changchun 130000, China

## Abstract

Parkinson's disease (PD), clinically characterized by motor and nonmotor symptoms, is a common progressive and multisystem neurodegenerative disorder, which is caused by both genetic and environmental risk factors. The main pathological features of PD are the loss of dopaminergic (DA) neurons and the accumulation of alpha-synuclein (*α*-syn) in the residual DA neurons in the substantia nigra pars compacta (SNpc). In recent years, substantial progress has been made in discovering the genetic factors of PD. In particular, a total of 19 PD-causing genes have been unraveled, among which some members have been regarded to be related to mitochondrial dysfunction. Mitochondria are key regulators of cellular metabolic activity and are critical for many important cellular processes including energy metabolism and even cell death. Their normal function is basically maintained by the mitochondrial quality control (MQC) mechanism. Accordingly, 1-methyl-4-phenyl-1,2,3,6-tetrahydropyridine (MPTP), a kind of neurotoxin, exerts its neurotoxic effects at least partially by producing its toxic metabolite, namely, 1-methyl-4-phenylpyridine (MPP+), which in turn causes mitochondrial dysfunction by inhibiting complex I and mimicking the key features of PD pathogenesis. This review focused on three main aspects of the MQC signaling pathways, that is, mitochondrial biogenesis, mitochondrial dynamics, and mitochondrial autophagy; hence, it demonstrates in detail how genetic and environmental factors result in PD pathogenesis by interfering with MQC pathways, thereby hopefully contributing to the discovery of novel potential therapeutic targets for PD.

## 1. Introduction

Parkinson's disease (PD) is the second most common neurodegenerative disorder after Alzheimer's disease (AD), from which over 1% of the population older than 60 years of age worldwide has suffered from related serious and even fatal illness [1]. The progressive loss of dopaminergic (DA) neurons and the accumulation of *α*-synuclein (*α*-syn) in the residual DA neurons in the substantia nigra pars compacta (SNpc) are the main pathological features of the disease [2]. The clinical features of PD are generally subdivided into motor and nonmotor symptoms. Motor symptoms mainly include muscle rigidity, bradykinesia, posture disorders, and resting tremors. These symptoms are traditionally considered to largely result from the loss of DA neurons in the SNpc [3]. Comparatively, the nonmotor symptoms of PD include depression, cognitive impairment, hallucinations, sleep disorders, olfactory disorders, and autonomic dysfunction. Besides the fact that some of these nonmotor symptoms may appear as early as one decade prior to the appearance of motor dysfunction [1], more intriguingly, some of these nonmotor symptoms in PD cannot be simply ascribed to the loss of DA neurons in the SNpc. As a matter of fact, the etiology of PD is yet to be fully defined, which is generally related to either genetic or environmental factors [4].

During the past two decades, substantial progress has been made in genetic mapping and understanding the roles of related genes in PD pathogenesis, especially single-gene causative genes. About 15% of the patients with PD have a family history, and 5-10% have been identified to have genetic susceptibility factors known as Mendelian forms [5]. To date, 19 pathogenic genes have been uncovered to be involved in PD pathogenesis, including 10 autosomal dominant genes and 9 autosomal recessive ones [6]. At present, SNCA (PARK1), LRRK2 (PARK8), CHCHD2 (PARK22), Parkin (PARK2), PINK1 (PARK6), and other gene mutations are widely studied in PD pathogenesis [7, 8].

**Figure 1:**
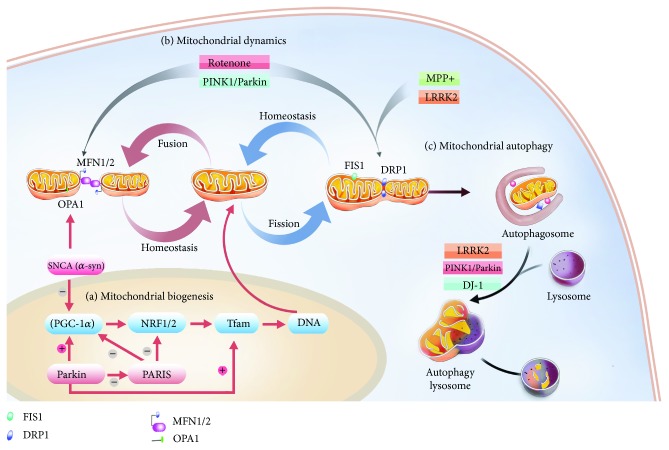
A schematic illustration of mitochondrial quality control in Parkinson's disease. (a) Mitochondrial biosynthesis plays an important role in mitochondrial quality control by creating new mitochondria to replace damaged mitochondria. (b) Mitochondrial dynamics include both mitochondrial division and mitochondrial fusion, which are critical for maintaining mitochondrial homeostasis and normal function. (c) Autophagy is generally a process by which cells degrade harmful or excessive cellular components and thus recycling components to maintain the homeostasis.

Although PD pathogenesis remains elusive, multiple essential processes have been found to contribute to the higher incidence among patients, including protein aggregation, impairment of the ubiquitin-proteasome pathway, oxidative stress, mitochondrial dysfunction, and neuroinflammation [9]. Accumulated evidence from PD models *in vitro* and *in vivo* suggested that mitochondrial dysfunction plays a major role in the pathogenesis of PD [8, 10–12]. The connection between mitochondrial dysfunction and PD was originally inspired by the administration of the neurotoxin 1-methyl-4-phenyl-1,2,3,6-tetrahydropyridine (MPTP), a by-product of the chemical synthesis of pethidine that may induce syndromes of PD [13, 14]. The neurotoxicity of MPTP is derived from its toxic metabolite 1-methyl-4-phenylpyridine (MPP+), which has a suppressive capacity over the electron transport chain by inhibiting the accumulation of complex I in the mitochondria, thus leading to mitochondrial dysfunction [15, 16]. Toxicants such as rotenone and paraquat, which are structurally similar to MPTP, further demonstrated the vital role of MPTP in mitochondrial dysfunction [17]. Given the indispensability of mitochondria within eukaryotic cells for energy metabolism, which is mainly driven by oxidative phosphorylation (OXPHOS), along with their involvements in many other physiological processes such as programmed cell death, innate immunity, autophagy, redox signaling, calcium homeostasis, and stem cell reprogramming, the role of mitochondria has received increasing attention during the pathogenesis of PD. Accordingly, its proper functioning is basically maintained by the mitochondrial quality control (MQC) machinery, a highly integrated network of signaling pathways, which is constantly involved in mitochondrial dynamics, biogenesis, and mitophagy [18]. Conversely, a variety of key biosynthetic processes such as ATP production, Ca^2+^ buffering, and apoptosis can be drastically undermined by impaired mitochondrial quality control pathways, which may in turn interfere with overall cellular homeostasis [19]. Reactive oxygen species (ROS) are by-products of biological aerobic metabolism, which include oxygen free radicals (such as superoxide anion radical (O_2_^·^) and hydroxyl radical (^·^OH)), nonradical oxidants (such as hydrogen peroxide (H_2_O_2_)), and oxygen-containing free radicals (such as nitric oxide (NO) and peroxyl radical (^·^OOH)) [20]. ROS are mainly produced by mitochondria, and maintaining low levels of ROS is critical for normal cellular function [21]. When the steady state equilibrium between ROS and the antioxidant defense system is destroyed, oxidative stress occurs, which not only causes harmful oxidation of biological macromolecules such as lipids, DNA, and proteins, but also causes the destruction of dopaminergic neurons [20–23]. Antioxidants include antioxidant enzymes (e.g., superoxide dismutase (SOD), catalase (CAT), glutathione peroxidase, and glutathione-S-transferase) and nonenzymatic antioxidant factors (e.g., melatonin, carotenoid and some microelements) [24]. Thus, ROS homeostasis plays a key role in maintaining the stability of mitochondrial quality control. Therefore, a detailed understanding of the precise role of the mitochondrial quality control pathways that underlie the pathogenesis of PD is conducive to the discovery of novel therapeutic targets for PD. In this review, we mainly focused on mitochondrial biogenesis, mitochondrial dynamics, and mitochondrial autophagy in order to gain a better understanding of the latest advances in mitochondrial quality control in PD pathogenesis, based on both genetic and environmental risk factors (see Figure 1).

## 2. Mitochondrial Biogenesis and PD

### 2.1. Mitochondrial Biogenesis

Mitochondrial biosynthesis plays an important role in mitochondrial quality control by creating new mitochondria to replace damaged mitochondria. Mammalian mitochondria are semiautonomous organelles containing products expressed from both mitochondrial genomes and nuclear genomes [25–27]. Despite the fact that the mitochondrial genome consists of circular double-stranded DNA (mtDNA), mitochondria still rely heavily on the expression of the nuclear genome to achieve most of its biological functions, possibly due to the limited coding capacity of mtDNA [28]. Mitochondrial biogenesis is activated by numerous different signals at the time of cellular stress or in response to environmental stimuli (nutrient availability, growth factors and hormones, toxins, temperature and oxygen fluctuations, among others) to form new mitochondria to maintain and restore mitochondrial structure, quantity, and function. Mitochondrial biogenesis is a complex and multistep cellular process, which not only involves the synthesis of either the inner or outer mitochondrial membrane but also involves the synthesis of mitochondrial-encoded proteins, the synthesis and import of nuclear-encoded mitochondrial proteins, and the replication of mtDNA [29]. Furthermore, the normal development of mitochondria requires coordinated expression of both the mitochondrial genome and the nuclear genome [30]. Currently, the mitochondrial biogenesis process is considered to be mainly regulated by peroxisome proliferator-activated receptor-gamma (PPAR*γ*) coactivator-1 alpha (PGC-1*α*) [31]. Adenosine monophosphate protein kinase (AMPK) and silent information regulator 1 (Sirt1) act as upstream regulators of PGC-1*α*, which activate PGC-1*α* by phosphorylation and deacetylation, respectively [32]. Upon activation of PGC-1*α* by phosphorylation or deacetylation, activated PGC-1*α* in turn activates nuclear respiratory factors 1 and 2 (NRF1 and NRF2), resulting in increased levels of NRF1 and NRF2 expression and their activities [33]. Subsequently, NRF1 and NRF2 activate mitochondrial transcription factor A (Tfam) to drive the transcription and replication of mitochondrial DNA, inducing mitochondrial biogenesis [34]. ROS, functioning as intracellular signaling messengers, play a key role in cell proliferation, apoptosis, and cellular oxidative damage [35]. Studies have shown that PGC-1*α* expression is regulated by ROS, thereby forming a potential network between PGC-1*α* and ROS [36–38]. It was found that NO can increase the expression of PGC-1*α* by activating AMPK and SIRT1, and H_2_O_2_ can also regulate the expression of PGC-1*α* through the AMPK pathway [32, 39]. At the same time, PGC-1*α* can also potently reduce the generation of mitochondrial-driven ROS, and loss of PGC-1*α* activity will lead to an increase in ROS [36, 40]. These regulatory factors play an important role in the maintenance of organelles and the expression of nuclear and mitochondrial genes required for biogenesis.

### 2.2. Abnormalities in Mitochondrial Biogenesis and Their Implications for PD

PGC-1*α* dysregulation affects mitochondrial biogenesis, leading to mitochondrial dysfunction, which will cause disease. Next, we will mainly discuss the relationship between PD and PGC-1*α* imbalance. A decrease in PGC-1*α* and the downregulation of various PGC-1*α* target genes were observed in DA neurons of PD [11, 41], suggesting that dysfunctional PGC-1*α* is involved in the clinical pathogenesis of PD. DA neurons in PGC-1*α* knockout mice are more sensitive to the neurodegenerative effects of MPTP and other stressors [36]. The importance of PGC-1*α* in the pathogenesis of PD was further revealed by the generation of PGC-1*α* deficient mice. PARIS is a transcriptional repressor that inhibits the expression of PGC-1*α* and its target gene NRF1 [42, 43]. PINK1/Parkin not only promotes mitochondrial biosynthesis by inducing the proteasomal degradation of PARIS to enhance PGC-1*α* transcription, but also directly interacts with Tfam to induce mtDNA replication and transcription of mitochondrial genes [44]. It has been widely accepted that the PINK1/Parkin gene acts as a major neuroprotective gene whose mutation is most likely to result in abnormal mitochondrial biogenesis. Besides, its mutation is the most common autosomal recessive form of PD. Quite a few studies have shown that *α*-syn binds to the PGC-1*α* promoter under oxidative stress and leads to PGC-1*α* suppression, for which mitochondrial biogenesis is in turn compromised [45]. In fact, it has been demonstrated in animal models that the inhibition of PGC-1*α* may sensitize experimental models to the neurodegenerative effects of MPTP, *α*-syn, and other stressors [36, 46], whereas the overexpression of PGC-1*α* has been shown to rescue either synaptic abnormalities caused by *α*-syn mutations or dopaminergic neuron loss induced by acute MPTP administration [36, 47, 48]. Studies have reported that PGC-1*α* is a broad and powerful regulator of ROS metabolism, and the expression of ROS antioxidant enzymes increases with the increase of PGC-1*α* [37–39]. Oxidative damage caused by the deletion of PGC-1*α* aggravates the degeneration of dopaminergic neurons [36]. Epidemiological studies have shown that high saturated fat diet is a risk factor for sporadic PD [49, 50]. The administration of palmitate to ICV in alpha-synuclein transgenic mice results in the hypermethylation of the PGC-1*α* promoter in the substantia nigra (SN), which in turn reduces PGC-1*α* gene expression and decreases mitochondrial content [51]. This further provides evidence that PGC-1*α* inhibition can promote sporadic PD. At the same time, research on PGC-1*α* is increasing in the search for PD treatment methods. cAMP response element binding protein (CREB) and activating transcription factor 2 (ATF2) are transcriptional activators of PGC-1*α*. Studies have found that metformin acts as a potential upstream regulator of mitochondrial gene transcription, stimulating PGC-1*α* promoter activity via the CREB and ATF2 pathways [52]. Collectively, previous studies indicated that PGC-1*α*, as a major regulator of mitochondrial biogenesis, is indeed a pivotal component involved in the pathogenesis of PD and may become a potential therapeutic target for PD.

## 3. Mitochondrial Dynamics and PD

### 3.1. Mitochondrial Dynamics

Mitochondria are dynamic organelles that are continuously undergoing fission and fusion in addition to organelle redistribution within the cytosol [53]. This property of mitochondria is collectively referred to as mitochondrial dynamics, which is essential for maintaining mitochondrial homeostasis and normal function. For instance, the length, shape, size, and number of mitochondria are basically controlled by their fusion and fission [54]. Mitochondria normally comprise the outer mitochondrial membranes (OMM) and the inner mitochondrial membranes (IMM), which constitute the border of the intermembrane space (IMS) and the matrix [55]. Mitochondrial fusion is a dynamic process in which two mitochondria not only fuse to form elongated mitochondria but also undergo component exchange, resulting in the renewal of the macromolecule as well as the ions [56, 57]. Furthermore, mitochondrial fusion requires a coordinated operation between the outer and inner membranes. In particular, mitochondrial fusion proteins in mammals are primarily composed of three members of the actin-related guanosine triphosphatase (GTPases) family, i.e., mitochondrial proteins (MFN) 1 and 2 and optic atrophy 1 (OPA1) [58]. MFN1 and MFN2 are involved in OMM fusion, while OPA1 is involved in IMM fusion [53, 59].

Mitochondrial division refers to the process of redistributing the mitochondrial matrix and mitochondrial DNA into two new mitochondria by separating the mitochondrial membrane, thereby isolating severely damaged mitochondria or protecting mitochondria against irreversible damages [60]. The dynein-related GTPase protein (DRP1) and mitochondrial fission (FIS1) are the major proteins responsible for fission [56].

Moreover, mitochondrial dynamics not only maintains the integrity of mitochondrial DNA and the balance of oxidative respiration, intracellular biosynthesis, and intracellular calcium signaling pathways but also underlies many essential processes, including neuronal remodeling and apoptosis. Imbalances of mitochondrial division and fusion often lead to structural alterations and dysfunction of mitochondria. Abnormalities in mitochondrial fusion often cause mitochondrial fragmentation, whereas the formation of megamitochondria usually results from defects in mitochondrial division. One of the most basic functions of mitochondrial fusion is the functional complementarity between mitochondria through the exchange of key components such as proteins from respiratory complexes as well as mtDNA [61–64]. Drastic alterations in mitochondrial fusion are most likely to lead to an increased mutation rate and genomic loss, which are definitely not conducive to maintaining the integrity of mtDNA [65].

### 3.2. Environmental Factors for Mitochondrial Dynamics

The kinetic defects within mitochondria usually become increasingly prominent during neurodegeneration, especially in the pathogenesis of PD [66, 67]. In particular, imbalances in the kinetic properties of neuronal mitochondria show strong association with PD through both environmental and genetic factors. For instance, an *in vitro* study using primary neurons showed that high concentrations of *rotenone* effectively induce mitochondrial division, whereas either exogenous overexpression of MFN1 or dominant inactivation of DRP1 results in a higher incidence of mitochondrial fusion, thus potentially preventing mitochondrial rupture as well as rescuing neurons from injury-induced dendrite degeneration and even neuronal death [68]. Similarly, Wang et al. established a PD model by MPP+ administration in order to determine the effect of MPP+ on mitochondrial dynamics. Their results have revealed that in neuron-derived SH-SY5Y cells, MPP+ accelerates mitochondrial fragmentation by increasing DRP1 expression levels and promoting the recruitment of DRP1 within mitochondria [69]. This study also showed that genetic inactivation of DRP1 completely blocks MPP+-induced mitochondrial fragmentation, and hence almost completely blocks downstream events such as MPP+-induced bioenergy homeostatic disruption, ROS production, and neuronal death, suggesting that DRP1-dependent mitochondrial fragmentation is mediated by MPP+-induced mitochondrial abnormalities. Excessive mitochondrial fragmentation is associated with the pathology of sporadic PD. Santos et al. demonstrated that only the inhibition of Drp1-induced fission and not Opa1-induced fusion rescues mitochondrial deficits in sporadic cases [70]. Thus, cellular dysfunction caused by kinetic defects within mitochondria plays a crucial role and may become a novel therapeutic target for PD.

### 3.3. Genetic Risk Factors for Mitochondrial Dynamics

In addition to toxins, specific mutations in the PD-related gene also play a role in the imbalance of mitochondrial dynamics. The *α*-syn protein is normally encoded by the SNCA gene, while alterations in the genetic locus of the SNCA gene have been found to encode dominant *α*-syn mutations (A53T, A30P, and E46K) besides having SNCA gene duplication and triplication. Furthermore, the overexpression of pathogenic *α*-syn (A53T or A30P) induces mitochondrial fragmentation by increasing the cleavage of OPA1 to inhibit mitochondrial fusion, which either MFN2 overexpression or DRP1 inhibition/elimination does not improve, suggesting that pathogenic *α*-syn-mediated mitochondrial fragmentation is possibly caused by defects in mitochondrial fusion/fission [71]. However, other studies have shown that, by synthesizing PINK1, Parkin, and DJ-1, fragmentation induced by pathogenic *α*-syn can be successfully rescued [72]. In addition, a recent study of rats overexpressing human A53T-*α*-synuclein (hA53T-*α*-syn) in the nigrostriatal pathway showed that, consistent with the findings of Guardia-Laguarta et al. [71], mitochondrial fragmentation induced by *α*-syn overexpression is at least partially reversed as well via the administration of small molecule mitochondrial division inhibitor-1 (mdivi-1) [73]. Nevertheless, whether mdivi-1 has a therapeutic potential for PD is poorly understood; hence, further exploration is needed.

Given that PINK1 and Parkin genes, as autosomal recessive genes, encode a mitochondrial serine/threonine protein kinase and a cytosolic E3 ubiquitin-protein ligase, respectively, they are currently regarded as being commonly associated with susceptibility to PD [74, 75]. Under normal conditions, the PINK1/Parkin signaling pathway regulates mitochondrial homeostasis by promoting DRP1-dependent mitochondrial division [76]. Based on the fact that MFN1, MFN2, and DRP1 are substrates for the ubiquitination of Parkin [77], mitochondrial fragmentation can be abolished simply by interfering with the calcium/calmodulin/calcineurin pathway, through which the involvement of Parkin signaling is indeed required for the dephosphorylation of DRP1 at serine 637 [78]. Moreover, the overexpression of PINK1/Parkin promotes mitochondrial division, resulting in an increase in the number of mitochondria, whereas the inactivation of PINK1/Parkin suppresses MFN ubiquitination, leading to the formation of megamitochondria [79]. The mutation of the PARK7 gene encoding DJ-1 is associated with the autosomal recessive form of early-onset PD [80, 81]. For instance, the loss of the normal DJ-1 function may result in mitochondrial fragmentation by an apparent decrease in the level of mitochondrial fusion. Conversely, mitochondrial rupture caused by DJ-1 deficiency is effectively rescued by the overexpression of PINK1/Parkin [82]. These findings suggested that DJ-1 is most likely to be directly involved in the PINK1/Parkin pathway, or at least regulates their corresponding activity. The LRRK2 mutation is one of the most common genetic factors for autosomal dominant parkinsonism, based on the fact that the LRRK2 mutant generally increases the level of mitochondrial DRP1 through mutual interaction with DRP1, thereby leading to severe mitochondrial rupture [83]. Together, alterations in mitochondrial dynamics are highly likely to be involved in a common pathogenic pathway for various genetic risk factors for PD, and may thus have great potential to become novel therapeutic goals.

## 4. Mitochondrial Autophagy and PD

### 4.1. Mitochondrial Autophagy

Autophagy is generally a process by which cells degrade harmful or excessive cellular components and thus recycle components to maintain homeostasis. Similarly, the removal of damaged mitochondria by autophagy is defined as mitochondrial autophagy (mitophagy) [84]. On the other hand, autophagy is also subdivided into three categories as follows: macroautophagy, microautophagy, and chaperone-mediated autophagy [85, 86]. Among them, macroautophagy is currently regarded as being the most essential subtype of autophagy, which is mainly composed of endoplasmic reticulum membranes, in order for the formation of cellular components, e.g. the cytoplasm, organelles, and protein aggregates. Thus, autophagosomes are basically a result of their recruitment, and then autophagosomes are normally transported to lysosomes for further degradation [87–89]. Autophagy can be induced by various forms of stress outside the cells such as starvation, growth factor deprivation, hypoxia, DNA damage, protein aggregates, damaged organelles, and intracellular pathogens [90, 91]. Autophagy can simply be subdivided into selective autophagy and nonselective autophagy depending on the selectivity of degraded subjects. Mitochondrial autophagy is a type of selective autophagy, meaning that mitochondria are selectively recruited into isolation membranes, which are sealed and then fused with lysosomes to eliminate the trapped mitochondria [92, 93]. Different steps of autophagy, including the amplification of the separation membrane and the production of autophagosomes, are mediated by autophagy-associated (Atg) proteins. More than 30 Atg proteins have so far been identified in yeast, among which Atg1-10, 12-14, 16, and 18 are regarded as “core Atg proteins,” and are hence required for autophagosome formation [94–96]. The autophagosome marker MAP1 light chain 3 (LC3; a homolog of yeast Atg8) in mammals is an ubiquitin-like protein covalently linked to phosphatidylethanolamine [97, 98]. LC3, normally located on the separating membrane and autophagosome, is definitively required for the formation of autophagosomes [99]. In yeast, Atg32 positioned on the OMM can be directly (the cytosolic domain of Atg32 contains a WXXL-like Atg8-binding motif) or indirectly (when bridged by Atg11) associated with Atg8 bound to the separation membrane to recruit mitochondria into the autophagosome [87, 100]. The homolog of Atg32 in mammals is BCL-2-like protein 13 (BCL2L13), which binds to LC3 during mitochondrial stress [101]. Mitochondrial autophagy is a type of macroautophagy that selectively removes damaged or nonessential mitochondria and hence plays an important role in mitochondrial quality control. Impaired mitochondrial autophagy disrupts mitochondrial function and results in the accumulation of defective organelles, inevitably leading to cell and tissue damages.

### 4.2. PINK1/Parkin Pathway and Mitochondrial Autophagy

Among the identified signaling pathways that underlie mitochondrial autophagy, the PINK1/Parkin pathway and receptor-mediated mitochondrial autophagy are more closely related to PINK1/Parkin [102]. The PINK1 protein encoded by PINK1 (PARK6) is a serine/threonine kinase, and the Parkin protein encoded by the Parkin (PARK2) gene is a RING finger containing the E3 ligase, which ubiquitinates many mitochondrial outer membrane proteins [75, 103].

Mitochondrial depolarization-induced mitochondrial autophagy is dependent on the PINK1/Parkin pathway, which is mediated by mitochondrial ubiquitination, which allows mitochondria-induced ubiquitination and adaptor proteins (p62, OPTN, and NDP52) to recognize each other and recruit adaptor proteins to mitochondria [104–107]. LC3 then recognizes and interacts with the adaptor protein to recruit ubiquitinated mitochondria to LC3-conjugated phagocytic cells (precursors of autophagosomes) to initiate autophagosome formation, and the depolarized mitochondria are ultimately degraded by lysosomal hydrolase [108]. Regarding normal mitochondrial function, PINK1 is expressed and introduced into the mitochondria and then rapidly passes through proteolysis; its expression level is maintained at a rather low level. When mitochondria are damaged, PINK1 proteolysis is inhibited, leading to the accumulation of PINK1 in damaged mitochondria, followed by specific recruitment of Parkin from the cytoplasm into damaged mitochondria in order for ubiquitinated mitochondria to initiate mitochondrial autophagy [109–112]. Therefore, mitochondrial depolarization, ROS production, and protein misfolding can trigger PINK1-mediated mitochondrial autophagy [113].

Receptor-mediated mitochondrial autophagy is mediated by mitochondrial autophagy receptors (BNIP3, NIX, and FUNDC1), and mitochondrial autophagy receptors localized on OMM interact directly with LC3 to mediate mitochondrial elimination [102]. Among them, Parkin-dependent ubiquitination of NIX and BNIP3 highlights the intricate crosstalk between receptor-mediated mitochondrial autophagy and the PINK1/Parkin pathway [108]. Mutations in PINK1 or Parkin cause defects in mitochondrial autophagy, and accumulation of damaged mitochondria causes oxidative stress and loss of nerve cells, which may be closely related to the pathogenesis of PD [107, 109, 114–116]. Chen et al. confirmed the role of Parkin and PINK1 in mitochondrial autophagy by the *α*-synuclein (A53T) transgenic mouse model [117]. When PINK1 or Parkin is deleted, these mice have increased the size and number of inclusion bodies, including neuronal inclusions of mitochondrial residual DA neurons and autophagosome, accumulated in the early stages prior to neurodegeneration, which further confirms the involvement of PINK1 and Parkin in mitochondrial clearance *in vivo* [117]. The PINK1/Parkin pathway is involved in mitochondrial autophagy, so neurons lacking PINK1 or Parkin are most likely to have defects in mitochondrial clearance and easily result in neuronal degeneration.

### 4.3. Other Genetic Risk Factors and Mitochondrial Autophagy

ERK signaling regulates mitochondrial autophagy, and DJ-1 activates ERK2 independently of the PINK1/Parkin pathway [118]. Previous studies have shown that the loss of DJ-1 leads to a decrease in basal autophagy, which is associated with decreased levels of phosphate-activated ERK2 [119]. The LRRK2 encoded by the PARK8 gene is a member of the leucine-rich repeat kinase family whose mutations are associated with autosomal dominant PD [120, 121]. Mutations in LRRK2 are a common cause of familial and sporadic PD [122]. Miro is an outer mitochondrial membrane protein, which serves to anchor mitochondria to microtubule motors [123, 124]. Mitochondria are highly mobile organelles whose movement should stop before mitochondrial autophagy begins [125]. In the early stages of clearance of damaged mitochondria, Miro is removed from the mitochondrial outer membrane, causing mitochondrial motion to cease, preparing for subsequent mitochondrial autophagy [126]. Some studies have previously shown that the PINK1/Parkin pathway induces Miro degradation and releases kinesins from mitochondria [125]. Others have shown that LRRK2 promotes the removal of Miro from damaged mitochondria by the formation of a complex with Miro, whereas the pathogenic LRRK2 mutation, mainly LRRK2G2019S, disrupts the structural integrity of the complex, thereby slowing Miro removal and causing mitochondrial stagnation as well as delaying subsequent mitochondrial autophagy [127].

### 4.4. Sporadic PD and Mitochondrial Autophagy

At present, the research on the mitochondrial autophagy of PD mainly focuses on familial PD, and there are few reports on sporadic PD and mitochondrial autophagy. Since sporadic PD accounts for 80%-85% of PD patients [128], it is particularly important to further explore the link between sporadic PD and mitochondrial autophagy. Many studies have already mentioned that Miro-related mitochondrial clearance disorders have a strong relationship with mutations in the PINK1/Parkin and LRRK2 genes. Recent studies have found that there is some correlation between mitochondrial autophagy and Miro in sporadic PD. Hsieh et al. found that Miro deficiency also causes mitochondrial autophagy defects in sporadic PD cases [127]. Studies have shown that lipid synthesis plays a role in PINK1-PARK2-mediated mitochondrial autophagy, and SREBF1, which is part of the lipogenesis pathway, has been shown to be a risk locus for sporadic PD [129]. Miro and SREBF1 link the pathogenesis of familial PD and sporadic PD, providing new ideas for exploring the pathogenesis of PD, especially the pathogenesis of sporadic PD. Miro and SREBF1 have also become potential targets for PD therapy. At the same time, more and more scientists have realized the importance of exploring the pathogenesis of sporadic PD for PD prevention and treatment, and more and more research will be done in this area.

## 5. Conclusions

Both environmental and genetic risk factors are involved in various aspects of mitochondrial quality control (mitochondrial biogenesis, kinetics, and autophagy) during the pathogenesis of PD. Although its complexity is not fully understood, recent studies have started to unravel the role of specific signaling pathways (e.g., the PINK1/Parkin pathway) in biosynthesis, kinetics, and autophagy during the regulation of mitochondrial quality control processes. This review summarized the current understanding of the mitochondrial quality control pathways that underlie the pathogenesis of Parkinson's disease and evaluated whether each signaling pathway and the related components could be potential targets of the prevention, diagnosis, and treatment of PD, based on both environmental and genetic risk factors for the mitochondrial quality control pathways at the forefront of translational research in PD. Hopefully, our study provides researchers with insightful opinions, and even points out promising general research directions, since each potential target has not been explained in detail in each section. Given the continuous progress in understanding the basic mechanism underlying the involvement of mitochondrial quality control pathways, it is widely believed that precision therapy in PD is most likely to precede breakthrough in the near future.
